# Supplementary material for: Sex–Gender Differences in Adult Coeliac Disease at Diagnosis and Gluten-Free-Diet Follow-Up
Source: Nutrients. 2022 Aug 4;14(15):3192. doi: 10.3390/nu14153192 (PMC9370199; doi:10.3390/nu14153192)
Supplement: Supplementary file 1 [file nutrients-14-03192-s001.zip › nutrients-1834299-supplementary.pdf]

Table S1. Features and comparison between pre-menopausal and post-menopausal female CD patients

	Pre-menopausal female patients n= 198	Post-menopausal female patients n= 32	***p-value***
**Median age at diagnosis**	34 (18-49)	55.5 (40-76)	***<0.0001***
**Median BMI* Kg/m^2 (range)**	21.3 (16.1-38.2)	22.5 (16.9-33.2)	0.39
**Comorbidities**			
Autoimmune	30.0%	34.4%	0.68
Non-autoimmune#	13.8%	21.9%	0.28
**Family history of CD§**	17.4%	18.7%	0.80
**≥3 years duration of symptoms/signs before the CD diagnosis**	41.9%	64.5%	***0.02***
**tTg IgA Ab ≥10 ULN**	51.7%	38.7%	0.24
**Clinical presentation**			
Classical symptoms	22.2%	21.9%	1
**GI° symptoms**			
Total of pts with GI symptoms	84.1%	90.6%	0.43
Nausea/Vomit	24.6%	31.2%	0.51
Heartburn	30.3%	40.6%	0.30
Regurgitation	23.1%	15.6%	0.49
Dysphagia	9.2%	15.6%	0.33
Postprandial fullness/early satiety	47.7%	46.8%	1
Abdominal pain	52.8%	50.0%	0.84
Abdominal bloating	61.0%	65.6%	0.69
Constipation	20.0%	25.0%	0.49
Diarrhoea	23.6%	31.2%	0.37
**Signs of malabsorption**	74.6%	59.4%	0.09
Anaemia	50.5%	35.5%	0.13
Hypoferritinaemia	66.3%	48.1%	0.30
Hypocholesterolaemia	8.7%	3.6%	0.70
Hypotriglyceridaemia	10.8%	7.1%	0.74
Hypoproteinaemia	5.0%	13.0%	0.14
**Osteopenia/Osteoporosis**	45.8%	71.4%	***0.01***
**Marsh 3C at diagnosis time**	57.1%	56.2%	1
**Lost at the FU**	12.5%	27.3%	0.08

***BMI**= Body Mass Index; **#Non-autoimmune comorbidities**= Metabolic, Cardiovascular; **§CD**= Celiac Disease; **°GI**= Gastrointestinal
